# New Appliances and Things Medical

**Published:** 1909-03-27

**Authors:** 


### NEW APPLIANCES AND THINGS MEDICAL.

[We shall be glad to receive at our Office, 28 & 29 Southampton Street, Strand, London, W.C., from the manufacturers, specimens of all new preparations and appliances.]

### LITHIO PIPERAZIN. IODOFORMIN.

(Dr. L. C. Marquart, Beuel, Bonn.)

We have received for notice the two preparations mentioned above. Lithio Piperazin is a combination of lithium salts with piperazin. The idea in making this substance was to obtain a drug which would combine the uric acid solvent action of piperazin with the milder solvent, and also diuretic properties of the lithium salts. The clinical results obtained with this substance appear to be favourable, increased urinary secretion and diminished urinary sediment along with increased excretion of uric acid having been noticed after its administration. It is prepared in tablets containing 7½ grains, and is given in doses of from 15 to 45 grains per diem. The second preparation, Iodoformin, is an odourless, white, dust-fine powder containing 75 per cent. of iodoform. It can be applied to gauze, wool, and ointments, which, unlike similar iodoform dressings, can be sterilised at a high temperature. When applied to wounds it acts like iodoform, since it contains 75 per cent. of the iodoform molecule unchanged.

### HETOL INJECTIONS.

(Kalle and Co., A. G. Biebrich, a Rhein.)

The work of Professor Lauderer upon the beneficial action of preparations of cinamic acid in tuberculosis has been brought before more than one congress. This treatment is being used at present at many sanatoria, and if the results of it in the hands of other observers are less markedly favourable than in those of Professor Lauderer himself, it must at least be admitted that no harm has ever come of the treatment; in the majority of cases suitably selected distinct improvement has been noted. Whether this improvement is due to a specific action of the cinamic acid upon the tuberculous process or to circulatory reaction, this is not the place to discuss. The substance now used for the subcutaneous or intra-venous injection of the patients is a sodium salt of cinamic acid, to which the name "hetol" has been given. Messrs. Kalle and Co., of Biebrich, have put up this substance in a very convenient form, which we have before us, consisting of a sterilised 1 per cent. solution, each tubule containing the quantity for one injection. The substance used for application to tuberculous ulcers by Professor Lauderer is a compound of cinamic acid and meta-kresol, forming a white crystalline powder, to which the name "hetokresol" has been given. Those interested in this treatment cannot do better than use these preparations.

### A SANATORIUM TEMPERATURE CHART.

The number of different patterns of temperature charts, as of midwifery forceps, is legion, but Messrs. Bale and Danielsson, of 83 Great Titchfield Street, have issued a sanatorium chart, based on a design from practical experience, which has a novel feature or two. It is intended for use as a compendious summary of the progress of a case for three months, and allowing for these limitations—only two temperature a day are noted—it should be of service.

### A REMINGTON TYPEWRITER WITH VISIBLE WRITING.

Users of the Remington typewriter, many of whom are medical men and scientists, have long struggled with the disadvantage of non-visible writing, simply because this machine, notwithstanding such a severe handicap, has always maintained its position as the leading writing machine. The appearance on the market of the latest model, No. 10 Remington, is therefore certain to receive a widespread welcome. We have seen and tried this new model, and are confident that it embodies perfect visibility of writing without sacrifice of more important features. The latest Remington typewriter also includes other important new features for promoting ease, convenience, and swiftness of operation. Since any new features incorporated in these machines are the fruit of careful study and experiment, and not of a mere straining after novelty, it is obvious that the makers' claim on behalf of their latest instrument, that it is foremost among typewriters, will be a difficult matter to dispute.

### MANUFACTURERS' EXHIBITS AT THE OPHTHALMIC EXHIBITION.

The Ophthalmic Exhibition held March 13 and 14 at the rooms of the Medical Society of London, 11 Chandos Street, Cavendish Square, was attended by a large number of ophthalmic surgeons and other medical practitioners. Of the firms showing novelties may be mentioned John Weiss and Son, Ltd., whose Bardsley needle-holders, amongst other ophthalmic instruments, were much appreciated. Martindale's showed the latest sterilised dressings, and A. Hawes, also of New Cavendish Street, showed many specimens of improved spectacles and lenses, including the new Inviso pinless spectacles. Leonard Liebemann, of Manchester, had on view a new ocular muscle-testing chart, whereby measurements are obtained entirely without mathematics. F. Davidson made a speciality of test cases and their new ophthalmoscope, and G. Martin of spectacle-frames. H. F. Angus and Co. showed the Baum Opthalmofundoscope, which gives a magnification equal to twice that of the usual patterns. C. W. Dixey and Son drew attention to an excellent lens-setting instrument and an improved trial-case. The Lenticular lens displayed by M. B. Bloom is a heavy minus or plus lens so ground that the weight is considerably reduced without detriment to its optical properties. A stigmatometer was shown by Sidney Richardson which is claimed to be the only instrument which will give the total refraction of the eye. G. Culver showed the Curtis motion-target; while in the exhibit of Down Bros. was a new syringe and various other novel and economical instruments.

